# Reversible cerebral Vasoconstriction syndrome intERnational CollaborativE (REVERCE) network: Study protocol and rationale of a multicentre research collaboration

**DOI:** 10.1177/23969873231182207

**Published:** 2023-06-17

**Authors:** Kristin Sophie Lange, So Youn Choi, Yu-Hsiang Ling, Shih-Pin Chen, Jérôme Mawet, Claire Duflos, Mi Ji Lee, Anne Ducros, Shuu-Jiun Wang, Alessandro Pezzini

**Affiliations:** 1 Department of Neurology, Charité – Universitätsmedizin Berlin, Berlin, Germany; 2 Center for Stroke Research Berlin (CSB), Charité – Universitätsmedizin, Berlin, Germany; 3 Department of Neurology, CHU Montpellier, Gui de Chauliac Hospital, Montpellier, France; 4 Department of Neurology, Seoul National University Hospital, Seoul National University College of Medicine, Seoul, Korea; 5 Department of Neurology, Neurological Institute, Taipei Veterans General Hospital, Taipei, Taiwan; 6 School of Medicine, College of Medicine, National Yang Ming Chiao Tung University, Taipei, Taiwan; 7 Brain Research Center, National Yang Ming Chiao Tung University, Taipei, Taiwan; 8 Division of Translational Research, Department of Medical Research, Taipei Veterans General Hospital, Taipei, Taiwan; 9 Institute of Clinical Medicine, College of Medicine, National Yang Ming Chiao Tung University, Taipei, Taiwan; 10 Emergency Headache Center, Department of Neurology, Lariboisière Hospital, Assistance Publique des Hôpitaux de Paris, Paris, France; 11 Clinical Research and Epidemiology Unit, Department of Public Health, CHU Montpellier, Montpellier University, Montpellier, France; 12 Charles Coulomb Laboratory, CNRS UMR5221, Montpellier University, Montpellier, France; 13 Department of Clinical and Experimental Sciences, Neurology Clinic, University of Brescia, Brescia, Italy

**Keywords:** Cerebrovascular disease, reversible cerebral vasoconstriction syndrome, headache, stroke, risk factors, prognosis, multicentric

## Abstract

**Introduction:**

Reversible cerebral vasoconstriction syndrome (RCVS) is a rare, but increasingly recognised cerebrovascular condition with an estimated annual age-standardised incidence of approximately three cases per million. Knowledge about risk factors and triggering conditions and information about prognosis and optimal treatment in these patients are limited.

**Methods:**

The REversible cerebral Vasoconstriction syndrome intERnational CollaborativE (REVERCE) project aims to elucidate the epidemiological and clinical characteristics of RCVS by collecting individual patient data from four countries (France, Italy, Taiwan and South Korea) in the setting of a multicentric study. All patients with a diagnosis of definite RCVS will be included. Data on the distribution of risk factors and triggering conditions, imaging data, neurological complications, functional outcome, risk of recurrent vascular events and death and finally the use of specific treatments will be collected. Subgroup analyses will be made based on age, gender, aetiology, ethnicity and geographical region of residence.

**Ethics and dissemination:**

Ethical approval for the REVERCE study will be obtained from national or local institutional review boards in the participating centres. When needed, a standardised data transfer agreement will be provided for participating centres. We plan dissemination of our results in peer-reviewed international scientific journals and through conference presentations. We expect that the results of this unique study will lead to better understanding of clinical and epidemiological characteristics of RCVS patients.

## Introduction

Reversible cerebral vasoconstriction syndrome (RCVS) is a clinico-radiological entity with reversible multifocal narrowing of the cerebral arteries, typically acute severe headache and sometimes neurological deficits or seizure. RCVS has been proposed as the unifying term for several conditions including Call-Fleming syndrome,^1^ benign angiopathy of the CNS,^2^ postpartum angiopathy, and drug-induced arteritis.^3^ Diagnostic criteria include angiographically confirmed, multifocal, segmental cerebral artery vasoconstriction, no evidence of aneurysmal SAH, a (near-) normal CSF profile, severe acute headaches (often with the characteristics of thunderclap headaches (TCH)^3^ with or without focal neurological deficits or seizures), and reversibility of angiographic findings within 12 weeks.^3–5^ Since publication of these criteria, it has been recognised that rarely, RCVS may occur without headache or in patients in whom information about headache is not available (e.g. due to coma, or severe aphasia).^6^ The clinical and radiologic spectrum of RCVS from pure cephalalgic forms to complications, including ischaemic and haemorrhagic lesions, and posterior encephalopathy syndrome (PRES).^7–13^ Prevalence of complications varies and is higher among cohorts from Western countries than in Asian cohorts.^7–11^ Despite a heterogeneous clinical presentation, the distinction between RCVS and its most feared differential diagnoses, namely primary angiitis of the central nervous system (PACNS) and vasospasm from aneurysmal SAH, is most often simple.^13–15^

RCVS is considered an increasingly recognised but still under-diagnosed condition. Based on the few population-wide data currently available, the estimated annual age-standardised incidence of cases leading to hospitalisation in US adults is 4.1 (95% CI, 3.5–4.8) cases per million in women versus 1.2 (95% CI, 0.9–1.6) cases per million in men, with an overall incidence of 2.7 (95% CI, 2.4–3.1) cases per million.^16^ In hospital-based series the disease incidence approximates 0.25% of patients presenting to a stroke unit and emergency headache clinic,^7,17^ 0.5% in headache clinics^2^ and 8.8% of patients presenting with TCH.^18^

Although vasoconstriction is reversible and the functional outcome reported by most studies is good,^8,19–21^ sequelae from neurological complications might impact the prognosis, with need for a referral to a rehabilitation clinic or nursing home in up to one-third of patients.^22^ Furthermore, recurrence of RCVS has been reported.^10,23^ Randomised controlled trials for treatment of RCVS have not been performed so far, and empirical treatment varies between countries. To date, information regarding causative risk factors, aetiology and long-term prognosis is scarce and mainly based on national cohorts. In addition, only few studies have taken ethnicity and geographical region of residence into account.^24^

There is a medical need to identify predictors for complications, for a poor functional outcome and for recurrence in order to guide clinical management of patients with RCVS. Given the relatively low incidence of the syndrome, only a multicentre study can provide a sufficiently large sample size to reach adequate statistical power for subgroup comparisons and longitudinal analyses. We therefore launched the REversible cerebral Vasoconstriction syndrome intERnational CollaborativE (REVERCE) project, an international consortium aimed at collecting individual patient data from neurological centres with specific expertise on RCVS.

## Methods and design

### The REVERCE network

**Figure 1.**
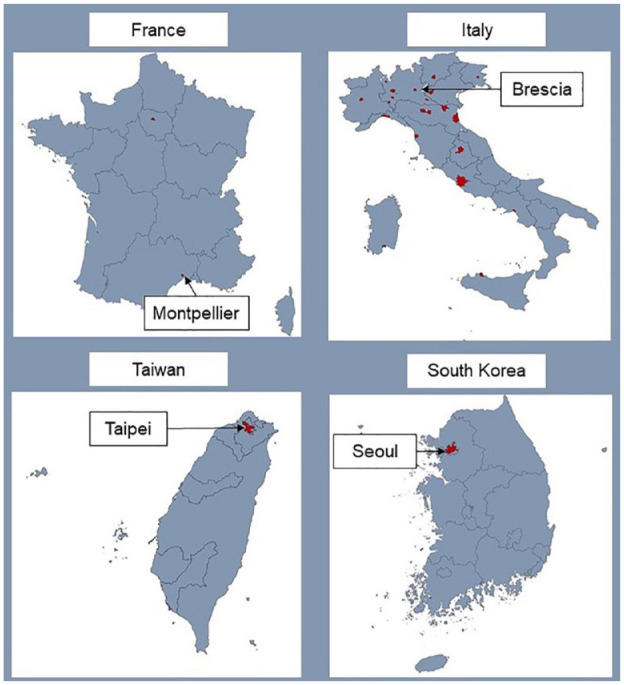
REVERCE recruiting centres. National coordinating centres are labelled with city names, participating centres are indicated in red, and are listed in the Supplemental Appendix.

REVERCE is an international investigator-driven network of neurological centres with special interest in headache and cerebral vascular disorders in adults, aimed at recruiting patients with RCVS in the setting of a hospital-based, multi-centre, observational study. The international coordinating centre is the University Hospital Montpellier, France. Each participating country has a national coordinating centre. A steering committee will be composed by two designated representatives from each participating country. There is no official chair and no official administration, board or secretary. To date the consortium is composed of referral centres located in the following four countries in Europe and Asia: France (two centres),^8,14,17,25–28^ Italy (28 centres, all referral centres participating in the Italian Project on Stroke at Young Age (IPSYS)),^7^ Taiwan (one centre)^2,10,29–32^ and South Korea (one centre)^9,33–37^ (Figure 1, Supplemental Appendix).

The REVERCE network welcomes participation and project proposals of further centres. The four national coordinating centres have established the REVERCE network with the first objective to compare characteristics of RCVS between European and Asian patients with data from the four largest European and Asian cohorts to date.^7–10^ In order to participate or suggest a novel project, the REVERCE coordinating centre in Montpellier can be reached via the corresponding author. On request, the standardised data collection form and project proposal form will be forwarded.

### Study aims and hypotheses

The Supplemental Table 1 lists selected intended projects, principal hypotheses and analysis plans.

The overall aim of REVERCE is to perform epidemiological and clinical research on RCVS, investigating risk factors and triggering conditions, pathophysiology, treatment, functional outcome, long-term risk of recurrent events and death, with special emphasis on ethnic and regional variation.

Our first project will be the comparison of patients with RCVS in Asia and Europe. Based on the published literature, we hypothesise that patients in Asia present less often neurological complications of RCVS, and less often secondary forms. If these hypotheses were confirmed with adjustment for selection bias, this could provide relevant insight into pathophysiology and the clinical spectrum of RCVS (subtypes).

Our second project will be the development of a clinical score to predict the risk for neurological complications of RCVS. This score could help clinicians to guide decisions on inpatient or outpatient care, length-of-stay, and use of intra-arterial procedures in severe cases. Based on results from national cohorts, we hypothesise that demographic variables (e.g. female sex, older age), comorbidities (e.g. depression or anxiety), and clinical characteristics (e.g. absence of TCH at onset) predict the risk for neurological complications.

### Patient eligibility

We will identify consecutive patients with ﬁrst-ever RCVS at institutions included in the REVERCE network, which serve as referral centres for neurological diseases in their region. Patients will be recruited both prospectively and retrospectively, according to the local study protocols. Retrospective patients had a qualifying event before the beginning of the study in each centre and will be identified through local registries. RCVS patients are recruited prospectively from the outpatient headache clinic, inpatient consultations, emergency unit, stroke unit or the neurology ward in each centre. Diagnostic criteria are summarised in Supplemental Table 2.

Only patients with definite RCVS according to the consensus criteria^3,4^ are included in the registry, regardless of whether they had neurological complications or not. In patients without (information about) headache, the consensus criteria for RCVS except for the presence of headache must apply, and occurrence of a focal deficit or seizure is necessary to determine the onset of RCVS. In ambiguous cases, the diagnosis must be validated by two members of the steering committee. Patients with probable RCVS, presenting either with headache probably attributed to RCVS according to the third edition of the International Classiﬁcation of Headache Disorders (ICHD-3),^5^ or with focal deficits, seizures, or vascular lesions suggestive of RCVS but with normal cerebral angiography do not qualify for inclusion.

### Data collection and storage

Participating centres will transfer their anonymised coded data electronically, according to current laws and legislation at each participating centre, to the REVERCE research team at the Montpellier University Hospital, France. Each participating centre will provide their data by using an encrypted file containing pre-specified variables of interest. The key linking anonymised data at the participating centres. All received data will be entered in a uniform database in IBM SPSS Statistics and stored on secured servers at the coordinating study centre with exclusive access to designated researchers involved in REVERCE. In case of incomplete or implausible data, the REVERCE research team will contact the study investigators to resolve these issues. All data will be processed, stored (for at least 15 years), and destroyed after end of the study according to European Union General Data Protection Regulation.

### Sample size and power calculation

Based on our literature search and consequently estimated available patient data, we aim to include at least 1000 patients for the first analyses, as this size will allow assessing epidemiological characteristics and prognosis of RCVS patients in meaningful subgroups.

### Baseline variables

Data for each individual patient are collected from the hospital at index event or at outpatient clinic evaluation. Baseline data include demographic characteristics, medical history and medication used on admission, potential precipitating and triggering factors, diagnostic workup (i.e. vascular and brain imaging, blood analyses, CSF analyses) and treatment. Cardiovascular risk factors and comorbidities as defined^38^ in Supplemental Table 3 will be documented.

### Precipitating factors for RCVS and triggers for headache episodes

We will collect detailed information on precipitating conditions and triggers for headache (Supplemental Table 4). In case of specific precipitating factors with a clear temporal association, RCVS will be qualified as *secondary* or *idiopathic*.^7–11^ A temporal association will generally be considered if the precipitating condition occurred within 4 weeks prior to RCVS onset, with exception for drugs and medications with a longer half-life. Postpartum RCVS mainly occurs during the first month after delivery,^39^ but might occur later as previously described for late onset postpartum eclampsia.^40^ Therefore, in RCVS occurring later than 4 weeks after delivery, postpartum will be considered as precipitating factor on a case-by-case basis and validated by two members of the steering committee. Remote emotional stress (e.g. serious illness of a family member, work overload) within 4 weeks prior to onset of RCVS will be documented, but not classified as a cause for secondary RCVS. If triggers for headache episodes (e.g. exertion, sexual activity) are reported, but there is no precipitating condition, RCVS is considered idiopathic.

### Clinical features

We will retain data on headache characteristics and any neurological symptoms. In particular, information on duration of symptoms prior to establishment of diagnosis, date of established diagnosis, presence of clinical complications (i.e. transient deficit (aura-like or transient ischaemic attack-like), persistent deficit, and seizure), and treatment will also be collected. At baseline, the severity of stroke (if any) is assessed with the National Institutes of Health Stroke Scale (NIHSS) score^41^ and the functional neurological outcome by the modified Ranking Scale (mRS).^42^

### Neuroimaging

At all centres, patients with suspected RCVS undergo cerebrovascular imaging (CTA, MRA or conventional cerebral angiography) and cerebral imaging (CT, MRI) applying the respective local imaging protocols. According to the consensus criteria for diagnosis of RCVS, cerebrovascular imaging is performed at least once at onset to confirm cerebral vasoconstriction with ⩾2 narrowing on two different arteries, and once at 3 months follow-up to confirm reversibility of vasoconstriction. Cerebral imaging is performed at onset and is repeated in case of clinical worsening. MRI sequences at all centres include at least DWI, ADC, FLAIR and T2* or SWI, respectively.

Cerebrovascular imaging will be assessed for (1) the presence of vasoconstriction on ⩾2 different arteries at onset, (2) the presence of concomitant cervical artery dissection, (3) the reversibility of vasoconstriction at 3 months follow-up.

Cerebral imaging will be evaluated for lesions considered as complications of RCVS, namely (1) ischaemic stroke (IS), (2) intracranial haemorrhage (intracerebral haemorrhage (ICH), subarachnoid haemorrhage (SAH), subdural haematoma (SDH)), and (3) PRES. We will document the delay from onset of symptoms to detection of radiological lesions.

These data will be provided by each centre, and will serve for analyses on the presence of the above-mentioned types of lesions.

For further in-depth analyses of radiological lesions, for example, location and size of lesions, we will establish a joint imaging database enabling for central qualitative reading of images following a standardised, structured protocol by experienced neuroradiologists or neurologists, who will be blinded to clinical data.

### Outcome

For retrospectively recruited patients, at least one follow-up at 3 months including proof of reversibility of headache and vasoconstriction is required. For prospectively recruited patients, follow-up evaluations are conducted systematically at 3 months for confirmation of the diagnosis, and subsequently according to the local study protocol. Primary outcomes measures will be the functional neurological status (mRS at 3 months), death and RCVS recurrence. As secondary outcomes for prospectively recruited patients, we will assess quality of life (European Quality of Life 5 Dimensions 5 Level Version, EQ-5D-5L),^43^ cognitive function (Montreal Cognitive Assessment, MoCA test),^44^ and emotional stress (Depression Anxiety Stress Scale, DASS-21)^45^ at 3 and 12 months follow-up.

### Current status of recruitment

For the first analyses from this network, patients recruited retrospectively and prospectively at the participating centres between September 2000 and February 2023 will be included. Currently, data on approximately 800 RCVS patients are available (≈35% of patients recruited from French centres, ≈10% from Italian, ≈40% from Taiwanese, and ≈15% from Korean centres, respectively).

### Lead of distinct project, authorship criteria and dissemination

The researcher(s) who would like to carry out a sub-project in the setting of the REVERCE network shall fill out a standardised project proposal form, which will then be reviewed and approved by the steering committee on the criteria of feasibility, scientific quality and a balanced attribution of sub-projects among the contributing centres. The researcher(s) who put forward the proposal take(s) the lead and will get first and senior authorships. Co-authorships are attributed taking into account all aspects important for the success of a research project. Study results will be published in peer-reviewed journals and be communicated to neurological communities by participating authors.

### Ethical considerations

At the coordinating centre, the University Hospital Montpellier, France, study approval for REVERCE was obtained from the local ethics committees (registration number IRB-MTP_2021_02_202100734). Each participating centre is responsible to obtain local ethics committee approval to collect and pool anonymised data with other centres, to fulfil local, regional and national legal and ethical requirements and to keep a local patient identifier.

### Statistical analysis

The statistical analysis plan specific to projects 1 and 2 is detailed in the Supplemental Table 1. We aim to perform statistical analyses with IBM SPSS Statistics or R (most recent versions). A *p* value of <0.05 with correction for multiple testing when appropriate and/or a 95% CI not containing the value 1 will be considered statistically significant. Differences between groups will be compared using ANOVA test, Student’s *t*-test for continuous variables and χ^2^ test for categorical variables. Univariate and multivariable logistic regression analysis will be performed for comparison between groups. In case of missing data at baseline, and if considered necessary, multiple imputation will be used. The cumulative risk of death and outcome events will be assessed with Kaplan–Meier survival analysis, and differences in survival between different subgroups by Log-Rank tests. We will use Cox regression models to obtain HRs and their corresponding 95% CIs to calculate the risk of death and outcome events while adjusting for confounders. Only patients for whom follow-up data is available will be included for these analyses, and patients who have died or were lost to follow-up will be censored from the last available follow-up.

## Future directions

We aim at prospectively enlarging the database by the contribution of the four participating countries and new collaboration partners. Beyond, REVERCE is intended to build the necessary infrastructure to (1) constitute a joint biobank to study the pathophysiology, genetic influences, and to identify possible biomarkers and therapeutic targets for RCVS and (2) perform randomised controlled trials for RCVS treatment with drug candidates including calcium channel blockers, sublingual nitroglycerin and/or treatment targeting Calcitonin Gene-Related Peptide (CGRP).

## Discussion

REVERCE aims to investigate the epidemiological and clinical aspects of RCVS, including risk factors and triggering conditions, to determine functional outcome and the risk of new or recurrent vascular events, and to study disease variation according to geographical region, continent, and ethnicity. The most important strength of our study is the participation of neurological centres from different countries and continents. REVERCE will be the largest study on RCVS with individual data sets ever conducted, with more than 800 patients already included. This large set of data provides sufficient statistical power to not only reliably quantify the differences in risk factors and precipitating conditions between men and women, different age groups, ethnic subgroups and possibly search for differences between region of residence, but also assess the risk of recurrent vascular events. The inclusion of highly phenotyped cases, the high level of completeness of data and the standardised data ascertainment are strengths of REVERCE.

Limitations include the risk of misclassification of risk factors and events of interest during follow-up, since retrospectively recruited patients’ data have been collected according to various not completely identical local protocols. We will harmonise variables across studies as much as possible. Since local registries were set up prior to REVERCE, they did not include all variables, which might implicate a risk of missing data. Furthermore, our study will cover a long time period in which data collection took place, possibly involving variability in the diagnostic workup because of differences in brain imaging protocols and improvements in imaging techniques.

In conclusion, REVERCE will provide sufficient patient numbers to allow for detailed description of the global distribution of risk factors and triggering conditions, and for the quantification of the cumulative risk of outcomes of patients with RCVS. The research protocol explicitly reaches out to other researchers and aims to become a platform that facilitates future collaborative research in the area of RCVS.

## Supplemental Material

sj-docx-1-eso-10.1177_23969873231182207 – Supplemental material for Reversible cerebral Vasoconstriction syndrome intERnational CollaborativE (REVERCE) network: Study protocol and rationale of a multicentre research collaboration Click here for additional data file. Supplemental material, sj-docx-1-eso-10.1177_23969873231182207 for Reversible cerebral Vasoconstriction syndrome intERnational CollaborativE (REVERCE) network: Study protocol and rationale of a multicentre research collaboration by Kristin Sophie Lange, So Youn Choi, Yu-Hsiang Ling, Shih-Pin Chen, Jérôme Mawet, Claire Duflos, Mi Ji Lee, Anne Ducros, Shuu-Jiun Wang and Alessandro Pezzini in European Stroke Journal

sj-docx-2-eso-10.1177_23969873231182207 – Supplemental material for Reversible cerebral Vasoconstriction syndrome intERnational CollaborativE (REVERCE) network: Study protocol and rationale of a multicentre research collaboration Click here for additional data file. Supplemental material, sj-docx-2-eso-10.1177_23969873231182207 for Reversible cerebral Vasoconstriction syndrome intERnational CollaborativE (REVERCE) network: Study protocol and rationale of a multicentre research collaboration by Kristin Sophie Lange, So Youn Choi, Yu-Hsiang Ling, Shih-Pin Chen, Jérôme Mawet, Claire Duflos, Mi Ji Lee, Anne Ducros, Shuu-Jiun Wang and Alessandro Pezzini in European Stroke Journal

sj-docx-3-eso-10.1177_23969873231182207 – Supplemental material for Reversible cerebral Vasoconstriction syndrome intERnational CollaborativE (REVERCE) network: Study protocol and rationale of a multicentre research collaboration Click here for additional data file. Supplemental material, sj-docx-3-eso-10.1177_23969873231182207 for Reversible cerebral Vasoconstriction syndrome intERnational CollaborativE (REVERCE) network: Study protocol and rationale of a multicentre research collaboration by Kristin Sophie Lange, So Youn Choi, Yu-Hsiang Ling, Shih-Pin Chen, Jérôme Mawet, Claire Duflos, Mi Ji Lee, Anne Ducros, Shuu-Jiun Wang and Alessandro Pezzini in European Stroke Journal

sj-docx-4-eso-10.1177_23969873231182207 – Supplemental material for Reversible cerebral Vasoconstriction syndrome intERnational CollaborativE (REVERCE) network: Study protocol and rationale of a multicentre research collaboration Click here for additional data file. Supplemental material, sj-docx-4-eso-10.1177_23969873231182207 for Reversible cerebral Vasoconstriction syndrome intERnational CollaborativE (REVERCE) network: Study protocol and rationale of a multicentre research collaboration by Kristin Sophie Lange, So Youn Choi, Yu-Hsiang Ling, Shih-Pin Chen, Jérôme Mawet, Claire Duflos, Mi Ji Lee, Anne Ducros, Shuu-Jiun Wang and Alessandro Pezzini in European Stroke Journal

sj-docx-5-eso-10.1177_23969873231182207 – Supplemental material for Reversible cerebral Vasoconstriction syndrome intERnational CollaborativE (REVERCE) network: Study protocol and rationale of a multicentre research collaboration Click here for additional data file. Supplemental material, sj-docx-5-eso-10.1177_23969873231182207 for Reversible cerebral Vasoconstriction syndrome intERnational CollaborativE (REVERCE) network: Study protocol and rationale of a multicentre research collaboration by Kristin Sophie Lange, So Youn Choi, Yu-Hsiang Ling, Shih-Pin Chen, Jérôme Mawet, Claire Duflos, Mi Ji Lee, Anne Ducros, Shuu-Jiun Wang and Alessandro Pezzini in European Stroke Journal

## References

[1] Call GK Fleming MC Sealfon S , et al. Reversible cerebral segmental vasoconstriction. Stroke 1988; 19: 1159–1170. 3046073 10.1161/01.str.19.9.1159

[2] Chen SP Fuh JL Lirng JF , et al. Recurrent primary thunderclap headache and benign CNS angiopathy: spectra of the same disorder? Neurology 2006; 67: 2164–2169. 17190937 10.1212/01.wnl.0000249115.63436.6d

[3] Calabrese LH Dodick DW Schwedt TJ , et al. Narrative review: reversible cerebral vasoconstriction syndromes. Ann Intern Med 2007; 146: 34–44. 17200220 10.7326/0003-4819-146-1-200701020-00007

[4] Ducros A . Reversible cerebral vasoconstriction syndrome. Lancet Neurol 2012; 11: 906–917. 22995694 10.1016/S1474-4422(12)70135-7

[5] Headache Classification Committee of the International Headache Society. The International Classification of Headache Disorders, 3rd edition (beta version). Cephalalgia 2013; 33: 629–808. 23771276 10.1177/0333102413485658

[6] Wolff V Ducros A . Reversible cerebral vasoconstriction syndrome without typical thunderclap headache. Headache 2016; 56: 674–687. 27016378 10.1111/head.12794

[7] Caria F Zedde M Gamba M , et al. The clinical spectrum of reversible cerebral vasoconstriction syndrome: the Italian project on stroke at young age (IPSYS). Cephalalgia 2019; 39: 1267–1276. 31060368 10.1177/0333102419849013

[8] Lange KS Forster O Mawet J , et al. Type of headache at onset and risk for complications in reversible cerebral vasoconstriction syndrome. Eur J Neurol 2022; 29: 130–137. 34390103 10.1111/ene.15064

[9] Choi HA Lee MJ Choi H , et al. Characteristics and demographics of reversible cerebral vasoconstriction syndrome: A large prospective series of Korean patients. Cephalalgia 2018; 38: 765–775. 28592180 10.1177/0333102417715223

[10] Chen SP Fuh JL Lirng JF , et al. Recurrence of reversible cerebral vasoconstriction syndrome: a long-term follow-up study. Neurology 2015; 84: 1552–1558. 25788554 10.1212/WNL.0000000000001473

[11] Singhal AB Hajj-Ali RA Topcuoglu MA , et al. Reversible cerebral vasoconstriction syndromes: analysis of 139 cases. Arch Neurol 2011; 68: 1005–1012. 21482916 10.1001/archneurol.2011.68

[12] Topcuoglu MA Kursun O Singhal AB . Coexisting vascular lesions in reversible cerebral vasoconstriction syndrome. Cephalalgia 2017; 37: 29–35. 26951336 10.1177/0333102416637826

[13] Muehlschlegel S Kursun O Topcuoglu MA , et al. Differentiating reversible cerebral vasoconstriction syndrome with subarachnoid hemorrhage from other causes of subarachnoid hemorrhage. JAMA Neurol 2013; 70: 1254–1260. 23939614 10.1001/jamaneurol.2013.3484

[14] de Boysson H Parienti JJ Mawet J , et al. Primary angiitis of the CNS and reversible cerebral vasoconstriction syndrome: A comparative study. Neurology 2018; 91: e1468–e1478. 30232250 10.1212/WNL.0000000000006367

[15] Rocha EA Topcuoglu MA Silva GS , et al. RCVS(2) score and diagnostic approach for reversible cerebral vasoconstriction syndrome. Neurology 2019; 92: e639–e647. 30635475 10.1212/WNL.0000000000006917

[16] Magid-Bernstein J Omran SS Parikh NS , et al. RCVS: symptoms, incidence, and resource utilization in a population-based US cohort. Neurology 2021; 97: e248–e253. 34050007 10.1212/WNL.0000000000012223 PMC8302148

[17] Ducros A Boukobza M Porcher R , et al. The clinical and radiological spectrum of reversible cerebral vasoconstriction syndrome. A prospective series of 67 patients. Brain 2007; 130: 3091–3101. 18025032 10.1093/brain/awm256

[18] Grooters GS Sluzewski M Tijssen CC . How often is thunderclap headache caused by the reversible cerebral vasoconstriction syndrome? Headache 2014; 54: 732–735. 24822246 10.1111/head.12256

[19] Katz BS Fugate JE Ameriso SF , et al. Clinical worsening in reversible cerebral vasoconstriction syndrome. JAMA Neurol 2014; 71: 68–73. 24190097 10.1001/jamaneurol.2013.4639

[20] Patel SD Topiwala K Saini V , et al. Hemorrhagic reversible cerebral vasoconstriction syndrome: A retrospective observational study. J Neurol 2021; 268: 632–639. 32894331 10.1007/s00415-020-10193-y

[21] John S Singhal AB Calabrese L , et al. Long-term outcomes after reversible cerebral vasoconstriction syndrome. Cephalalgia 2016; 36: 387–394. 26088331 10.1177/0333102415591507

[22] Patel SD Topiwala K Otite Oliver F , et al. Outcomes among patients with reversible cerebral vasoconstriction syndrome: a nationwide United States analysis. Stroke 2021; 52: 3970–3977. 34470494 10.1161/STROKEAHA.121.034424

[23] Boitet R de Gaalon S Duflos C , et al. Long-term outcomes after reversible cerebral vasoconstriction syndrome. Stroke 2020; 51: 670–673. 31842705 10.1161/STROKEAHA.119.027703

[24] Song TJ Lee KH Li H , et al. Reversible cerebral vasoconstriction syndrome: a comprehensive systematic review. Eur Rev Med Pharmacol Sci 2021; 25: 3519–3529. 34002826 10.26355/eurrev_202105_25834

[25] Ducros A Fiedler U Porcher R , et al. Hemorrhagic manifestations of reversible cerebral vasoconstriction syndrome: frequency, features, and risk factors. Stroke 2010; 41: 2505–2511. 20884871 10.1161/STROKEAHA.109.572313

[26] Mawet J Boukobza M Franc J , et al. Reversible cerebral vasoconstriction syndrome and cervical artery dissection in 20 patients. Neurology 2013; 81: 821–824. 23884040 10.1212/WNL.0b013e3182a2cbe2

[27] Ducros A Wolff V . The typical thunderclap headache of reversible cerebral vasoconstriction syndrome and its various triggers. Headache 2016; 56: 657–673. 27015869 10.1111/head.12797

[28] Lange KS Tuloup G Duflos C , et al. Complications of reversible cerebral vasoconstriction syndrome in relation to age. J Neurol. Epub ahead of print 2023. DOI: 10.1007/s00415-023-11708-z. 10.1007/s00415-023-11708-z PMC10267248 37052670

[29] Chen SP Fuh JL Wang SJ , et al. Magnetic resonance angiography in reversible cerebral vasoconstriction syndromes. Ann Neurol 2010; 67: 648–656. 20437562 10.1002/ana.21951

[30] Chen SP Wang SJ . Pathophysiology of reversible cerebral vasoconstriction syndrome. J Biomed Sci 2022; 29: 72. 36127720 10.1186/s12929-022-00857-4 PMC9489486

[31] Chen SP Chang YA Chou CH , et al. Circulating microRNAs Associated With Reversible Cerebral Vasoconstriction Syndrome. Ann Neurol 2021; 89: 459–473. 33314303 10.1002/ana.25965

[32] Chen SP Wang SJ . Hyperintense vessels: an early MRI marker of reversible cerebral vasoconstriction syndrome? Cephalalgia 2014; 34: 1038–1039. 24715501 10.1177/0333102414529193

[33] Cho S Ling YH Lee MJ , et al. Temporal profile of blood-brain barrier breakdown in reversible cerebral vasoconstriction syndrome. Stroke 2020; 51: 1451–1457. 32299322 10.1161/STROKEAHA.119.028656

[34] Cho S Lee MJ Chung CS . Effect of nimodipine treatment on the clinical course of reversible cerebral vasoconstriction syndrome. Front Neurol 2019; 10: 644. 31275233 10.3389/fneur.2019.00644 PMC6591369

[35] Cho S Lee MJ Gil YE , et al. RCVS-TCH score can predict reversible cerebral vasoconstriction syndrome in patients with thunderclap headache. Sci Rep 2021; 11: 7750. 33833341 10.1038/s41598-021-87412-7 PMC8032806

[36] Choi HA Lee MJ Chung CS . Cerebral endothelial dysfunction in reversible cerebral vasoconstriction syndrome: a case-control study. J Headache Pain 2017; 18: 29. 28229321 10.1186/s10194-017-0738-x PMC5321640

[37] Lee MJ Cha J Choi HA , et al. Blood-brain barrier breakdown in reversible cerebral vasoconstriction syndrome: Implications for pathophysiology and diagnosis. Ann Neurol 2017; 81: 454–466. 28195428 10.1002/ana.24891

[38] Kernan WN Ovbiagele B Black HR , et al. Guidelines for the prevention of stroke in patients with stroke and transient ischemic attack: a guideline for healthcare professionals from the American Heart Association/American Stroke Association. Stroke 2014; 45: 2160–2236. 24788967 10.1161/STR.0000000000000024

[39] Skeik N Porten BR Kadkhodayan Y , et al. Postpartum reversible cerebral vasoconstriction syndrome: review and analysis of the current data. Vasc Med 2015; 20: 256–265. 25835347 10.1177/1358863X14567976

[40] Minnerup J Kleffner I Wersching H , et al. Late onset postpartum eclampsia: it is really never too late - a case of eclampsia 8 weeks after delivery. Stroke Res Treat 2010; 2010: 798616. 20798839 10.4061/2010/798616 PMC2925215

[41] Brott T Adams HP Jr Olinger CP , et al. Measurements of acute cerebral infarction: a clinical examination scale. Stroke 1989; 20: 864–870. 2749846 10.1161/01.str.20.7.864

[42] van Swieten JC Koudstaal PJ Visser MC , et al. Interobserver agreement for the assessment of handicap in stroke patients. Stroke 1988; 19: 604–607. 3363593 10.1161/01.str.19.5.604

[43] Rabin R de Charro F . EQ-5D: a measure of health status from the EuroQo l Group. Ann Med 2001; 33: 337–343. 11491192 10.3109/07853890109002087

[44] Nasreddine ZS Phillips NA Bedirian V , et al. The Montreal Cognitive Assessment, MoCA: a brief screening tool for mild cognitive impairment. J Am Geriatr Soc 2005; 53: 695–699. 15817019 10.1111/j.1532-5415.2005.53221.x

[45] Lovibond PF Lovibond SH . The structure of negative emotional states: comparison of the Depression Anxiety Stress Scales (DASS) with the Beck Depression and anxiety inventories. Behav Res Ther 1995; 33: 335–343. 7726811 10.1016/0005-7967(94)00075-u

