# Supplementary material for: Reversible cerebral Vasoconstriction syndrome intERnational CollaborativE (REVERCE) network: Study protocol and rationale of a multicentre research collaboration
Source: Eur Stroke J. 2023 Jun 17;8(4):1107–13. doi: 10.1177/23969873231182207 (PMC10683719; doi:10.1177/23969873231182207)
Supplement: sj-docx-1-eso-10.1177_23969873231182207 – Supplemental material for Reversible cerebral Vasoconstriction syndrome intERnational CollaborativE (REVERCE) network: Study protocol and rationale of a multicentre research collaboration [file sj-docx-1-eso-10.1177_23969873231182207.docx]

**APPENDIX**

**Reversible cerebral Vasoconstriction syndrome intERnational CollaborativE (REVERCE) network: study protocol and rationale of a multicentre research collaboration**

**Members of the REVERCE Network**

*France:* Headache Unit, Neurology Department, CHU Montpellier, Montpellier (Anne Ducros, Kristin Sophie Lange), Stroke Unit, Neurology Department, CHU Montpellier, Montpellier (Lucas Corti, Caroline Arquizan), Clinical Research and Epidemiology Unit, Department of Public Health, CHU Montpellier, Montpellier (Claire Duflos); Emergency Headache Centre, Neurology Department, Lariboisière Hospital, APHP, Paris (Jérôme Mawet, Caroline Roos, Cécilia Burcin, Gabrielle Tuloup); Neurovascular Unit, Neurology Department, Lariboisière Hospital, APHP, Paris (Peggy Reiner, Claire Gobron)

*Taiwan:* Department of Neurology, Neurological Institute, Taipei Veterans General Hospital, Taipei (Shuu-Jiun Wang, Jong-Ling Fuh, Shih-Pin Chen, Yen-Feng Wang, Yu-Hsiang Ling).

*South Korea:* Department of Neurology, Seoul National University Hospital, Seoul National University College of Medicine, Seoul (current affiliation of Mi Ji Lee and So Youn Choi). Department of Neurology, Uijeongbu Eulji Medical Center, Eulji University School of Medicine, Uijeongbu (Soohyun Cho). The data were collected from Department of Neurology, Samsung Medical Center, Sungkyunkwan University School of Medicine, Seoul (Mi Ji Lee, So Youn Choi, Chin-Sang Chung, Soohyun Cho).

*Italy*: SC Neurology, AUSL, IRCCS Reggio Emilia, Reggio Emilia (Marialuisa Zedde); Stroke Unit, Vascular Neurology, ASST Spedali Civili, Brescia (Massimo Gamba); UO Cerebrovascular Diseases, IRCCS ‘‘Carlo Besta’’ Neurologic Insitute, Milan (Anna Bersano); Stroke Unit, Sant’Andrea Hospital, University` ‘‘La Sapienza’’, Rome (Maurizia Rasura); Stroke Centre, Department of Neurology, Sacro Cuore Negrar Hospital, Verona (Alessandro Adami); UOC Neurology, ‘‘San Giovanni-Addolorata’’ Hospital, Rome (Carlo Piantadosi); Rehumatology Clinic, Department of Medicine, ‘‘Santa Maria della Misericordia’’ University Hospital, Udine (Luca Quartuccio); UO Neurology, Department of Neuroscience and Rehabilitation, Ferrara University Hospital, Ferrara (Cristiano Azzini); SC Neurology and Stroke Unit, Department of Neuroscience and Rehabilitation, ‘‘G. Brotzu’’ Hospital, Cagliari (Maurizio Melis); UO Neurology, University of Insubria, Varese (Maria Luisa Delodovici); Departemnt of Medicine, UOC Neurology, ASST Pavia, Voghera (Carlo Dallocchio); Department of Neuroscience, Rehabilitation, Ophtalmology, Genetics and Pediatric Sciences, University of Genoa, Genoa (Carlo Gandolfo); Department of Neuroscience, Stroke Unit, University of Turin (Paolo Cerrato); Stroke Unit, Department of Neurological Sciences, ‘‘Niguarda Ca’ Granda’’ Hospital, Milan (Cristina Motto); SS Neurovascular Ospedale Maria Vittoria, ASL City of Turin, Turino (Fabio Melis); UO Neurology, Pisa University Hospital, Pisa (Alberto Chiti); UOC Neurology and Metropolitan Stroke Network, IRCCS Neurological Sciences Bologna, Maggiore Hospital, Bologna (Mauro Gentile, Andrea ZIni); Stroke Unit, UO Neurology, “S. Chiara’’ Hospital, Trento (Valeria Bignamini); UOC Neurology, AUSL Romagna, Ravenna (Enrico Maria Lotti); UOC Neurology, ‘‘San Giovanni di Dio e Ruggi d’Aragona’’ University Hospital, Salerno (Antonella Toriello); Stroke Unit, Department of Neuroscience, “Carlo Poma” Hospital, Mantova (Giorgio Silvestrelli); Stroke Unit and Cardiovascular Medicine, University of Perugia (Maurizio Paciaroni); Center for Thrombosis and Haemostasis, Humanitas Clinical and Research Centre, IRCCS, Rozzano-Milano (Corrado Lodigiani); Neurological Emergencies and Stroke Unit, Humanitas Clinical and Research Centre, IRCCS, Rozzano-Milano (Simona Marcheselli); Department of Neurology, ‘‘Madonna del Soccorso’’ Hospital, San Benedetto del Tronto (Sandro Sanguigni); UO Neurology, IRCCS "San Martino" Hospital, Genoa (Massimo Del Sette); Stroke Unit, Civic Hospital, Palermo (Serena Monaco); Department of Neurology, Saarland University Medical Centre, Homburg (Piergiorgio Lochner); UOC Neurology, ASST Melegnano-Martesana, Vizzolo Predabissi (Carla Zanferrari); Stroke Unit, Department of Emergencies, ‘‘S. Camillo-Forlanini’’ Hospital, Rome (Sabrina Anticoli); Department of Clinical and Experimantal Sciences, Neurology Clinic, University of Brescia, Brescia (Alessandro Pezzini)
