# Supplementary material for: Reversible cerebral Vasoconstriction syndrome intERnational CollaborativE (REVERCE) network: Study protocol and rationale of a multicentre research collaboration
Source: Eur Stroke J. 2023 Jun 17;8(4):1107–13. doi: 10.1177/23969873231182207 (PMC10683719; doi:10.1177/23969873231182207)
Supplement: sj-docx-2-eso-10.1177_23969873231182207 – Supplemental material for Reversible cerebral Vasoconstriction syndrome intERnational CollaborativE (REVERCE) network: Study protocol and rationale of a multicentre research collaboration [file sj-docx-2-eso-10.1177_23969873231182207.docx]

**Supplementary Table 1. List of selected intended projects, principal hypotheses and analysis plans**

| **Project** | **#1: Comparison of the clinico-radiological spectrum of RCVS in Europe and Asia** |
| --- | --- |
| Principal Hypotheses | 1) Patients in Asia are less likely to present neurological complications from RCVS compared to patients in Europe  2) Secondary forms of RCVS are less frequent in Asia compared to Europe |
| Statistical Analysis | For comparison of Asian and European patients, categorical variables will be compared by the Pearson's chi-squared test. For continuous variables, Student’s t-test will be applied for comparison of sufficiently normally distributed data and the Mann−Whitney U-test for non-normally distributed data.  Primary endpoint is the occurrence of neurological complications (combined endpoint of IS, intracranial haemorrhage, PRES).  For the first hypothesis, secondary endpoints are each type of complication, combinations of neurological complications per pathophysiology (BBB breakdown-related: haemorrhage and PRES, vasocontriction-related: IS), and combination of clinico-radiological complications (focal deficits, seizure, IS, intracranial haemorrhage, PRES). For the second hypothesis, endpoint is the aetiology of RCVS (idiopathic vs. secondary).  For univariable analysis, we will indicate odds ratios (OR) and their 95% confidence intervals (CIs) from Pearson's chi-squared test. To determine independent associations of the geographical region and endpoints, we will perform a stepwise forward logistic regression for estimation of multivariable OR and their 95% CI, with adjustment for variables known to be associated with the endpoints, and adjustment for centre and recruitment mode (e.g., headache centre or stroke unit) to adjust for selection bias. |
| **Project** | **#2: Prediction of neurological complications from RCVS using demographic and clinical variables** |
| Principal Hypotheses | 1) Demographic factors, comorbidities and clinical presentation at RCVS onset are associated with the risk of neurological complications in RCVS  2) A prediction score for risk of neurological complications is able to identify patients at a high risk for complications |
| Statistical Analysis | 1) Development of a clinical prediction model for the risk of neurological complications in RCVS: In a derivation set including 50% of patients from each sub-cohort (French, Italian, Taiwanese and Korean cohorts), we will perform univariable logistic regression analysis of demographic factors, comorbidities and clinical characteristics associated with a combined endpoint of neurological complications from RCVS (IS, intracranial haemorrhage, PRES). Secondary endpoints are each type of complication, combinations of neurological complications per pathophysiology (BBB breakdown-related: haemorrhage and PRES, vasocontriction-related: IS), and combination of clinico-radiological complications (focal deficits, seizure, IS, intracranial haemorrhage, PRES). We will select a list of candidate predictors for complications which were significant in univariable logistic regression with a p value of <0.20. Multivariable forward stepwise regression models will be developed to assign a score value to each selected variable, after removing those variables no longer associated with risk (p>0.05). The maximum number of variables per model will be adapted depending on the events per variable to avoid overfitting. Different combinations of predictive variables will be used to generate potential risk scores by summing up the total number of weighted risk factors, with the risk factor weighting schemes from each score tested individually. C statistics for these potential scores will be calculated and the score with the highest c statistic (best prediction) will be selected, based on the closest integer of the beta coefficient. The diagnostic performance of the score will be assessed by its discriminative power using the concordance index (C-index). We will calculate specificity, sensitivity, and positive and negative predictive values by analysis of the receiver operating characteristic curve (ROC). Calibration power will be assessed by the Hosmer and Lemeshow goodness-of-fit test or index of prediction accuracy. The Youden index will be used to determine the optimal cut-off score, and we will calculate its accuracy and likelihood based on the sensitivity and specificity.  2) Validation of a clinical risk score for neurological complications in RCVS: The remaining 50% of each sub-cohort cohorts will serve as an independent validation set. Using this validation set, the diagnostic performance of the risk prediction score will be validated. |
| **Project** | **#3: Prediction of RCVS outcome** |
| Objectives | 1) To identify predictors of an unfavourable functional outcome, defined as an mRS ≥2 at 3 months follow-up.  2) To identify predictors of death within 3 months of RCVS onset.  3) To assess quality of life (EQ-5D-5L), cognitive function (MoCA-test) and level of emotional stress (DASS-21) at 3 months follow-up. |
| **Project** | **#4: Prediction of RCVS recurrence** |
| Objectives | To identify demographic, clinical and radiological predictors of long-term recurrence of RCVS. |
| **Project** | **#5: Seasonal influences on occurrence of RCVS** |
| Objectives | To investigate whether meteorological factors correlate with the occurrence of RCVS. |
| **Project** | **#6: Pathophysiology and Biomarkers for RCVS** |
| Objectives | 1) To identify genetic variants predisposing for RCVS  2) To identify biomarker candidates able to discriminate RCVS from other primary and secondary headache disorders, and other causes of intracranial stenoses  3) To assess the role of endothelial dysfunction in RCVS  4) To identify and confirm potential therapeutic targets (e.g., CGRP) |
| **Project** | **#7: Treatment for RCVS** |
| Objectives | To assess the efficacy (amelioration of headache, amelioration of vasospasm, prevention of neurological complications), the safety and tolerability of drug candidates for RCVS, including calcium channel blockers, sublingual nitroglycerin and/or treatment targeting CGRP. |

Abbreviations: BBB, blood-brain barrier; CGRP, Calcitonin Gene-Related Peptide; IS, ischaemic stroke; mRS, modified Rankin Scale; PRES, posterior reversible encephalopathy syndrome; RCVS, reversible cerebral vasoconstriction syndrome.
