# Supplementary material for: Reversible cerebral Vasoconstriction syndrome intERnational CollaborativE (REVERCE) network: Study protocol and rationale of a multicentre research collaboration
Source: Eur Stroke J. 2023 Jun 17;8(4):1107–13. doi: 10.1177/23969873231182207 (PMC10683719; doi:10.1177/23969873231182207)
Supplement: sj-docx-3-eso-10.1177_23969873231182207 – Supplemental material for Reversible cerebral Vasoconstriction syndrome intERnational CollaborativE (REVERCE) network: Study protocol and rationale of a multicentre research collaboration [file sj-docx-3-eso-10.1177_23969873231182207.docx]

**Supplementary Table 2. Diagnostic criteria for definite RCVS.**

| **Criteria** | **Patients with headache** | **All patients** | **Patients without headache** |
| --- | --- | --- | --- |
| **Consensus criteria**  **for RCVS** | • Acute and severe headache (often thunderclap headache with typical triggers^5^) with or without focal deficits or seizures†  • Complete or substantial normalisation of arteries shown by follow-up indirect or direct angiography or transcranial doppler within 12 weeks of clinical onset | • Uniphasic course without new symptoms more than  1 month after clinical onset^5^  • Segmental vasoconstriction of cerebral arteries shown by indirect (eg, magnetic resonance or CT) or direct catheter angiography  • No evidence of aneurysmal SAH  • Normal or near-normal CSF (protein concentrations <100 mg/dL, <15 white blood cells per μL) | • Focal deficits or seizures†  • Not better accounted for by another secondary headache disorder  • Complete normalisation of arteries shown by follow-up indirect or direct angiography within 12 weeks of clinical onset |

Adapted from the criteria proposed by Calabrese et al. [3], Ducros [4] and from the International Headache Society [5]. †Onset of headache, seizure or focal deficit determines onset of RCVS. SAH, subarachnoid haemorrhage.
