# Supplementary material for: Reversible cerebral Vasoconstriction syndrome intERnational CollaborativE (REVERCE) network: Study protocol and rationale of a multicentre research collaboration
Source: Eur Stroke J. 2023 Jun 17;8(4):1107–13. doi: 10.1177/23969873231182207 (PMC10683719; doi:10.1177/23969873231182207)
Supplement: sj-docx-4-eso-10.1177_23969873231182207 – Supplemental material for Reversible cerebral Vasoconstriction syndrome intERnational CollaborativE (REVERCE) network: Study protocol and rationale of a multicentre research collaboration [file sj-docx-4-eso-10.1177_23969873231182207.docx]

**Supplementary Table 3. Baseline demographic variables, risk factors and diseases.**

| **Variable** | **Definition** |
| --- | --- |
| **Demographic characteristics** | |
| Age | Male/female |
| Sex | Asian, European, other |
| Ethnicity |  |
| **Cardiovascular risk factors** | |
| Hypertension | - Hypertension either in the patients’ medical history, or identified during admission for the index event after the acute phase - Use of antihypertensive medication and/or systolic blood pressure of ≥140 mm Hg and/or diastolic blood pressure of ≥90 mm Hg |
| Diabetes | - Diabetes either in the patients’ medical history, or identified during admission for the index event - Use of diabetic medication and/or a fasting plasma glucose >7 mmol/L and/or 2 hours plasma glucose ≥11.1 mmol/L during oral glucose tolerance test (OGTT) and/or HbA1C≥6.5% (48 mmol/mol) and/or symptoms of hyperglycaemia or hyperglycaemic crisis and a random glucose >11.1 mmol/L |
| Hypercholesterolaemia | - Hypercholesterolaemia either in the patients’ medical history, or identified during admission for the index event - Use of statins and/or fasting total cholesterol level ≥5.2 mmol/L (200 mg/dL) |
| Active smoking | - Any current smoking |
| **Disease state** | |
| Migraine | - Migraine either in the patients’ medical history, or identified during admission for the index event - Defined according to the ICHD-3 criteria |
| Depression or anxiety | - Depression or anxiety either in the patients’ medical history, or identified during admission for the index event |
