# Supplementary material for: Reversible cerebral Vasoconstriction syndrome intERnational CollaborativE (REVERCE) network: Study protocol and rationale of a multicentre research collaboration
Source: Eur Stroke J. 2023 Jun 17;8(4):1107–13. doi: 10.1177/23969873231182207 (PMC10683719; doi:10.1177/23969873231182207)
Supplement: sj-docx-5-eso-10.1177_23969873231182207 – Supplemental material for Reversible cerebral Vasoconstriction syndrome intERnational CollaborativE (REVERCE) network: Study protocol and rationale of a multicentre research collaboration [file sj-docx-5-eso-10.1177_23969873231182207.docx]

**Supplementary Table 4.** **Precipitating conditions and headache triggers in RCVS.**

| **Precipitating conditions** | **Headache triggers** |
| --- | --- |
| **Physiologic conditions** | Sexual activity |
| Pregnancy | Exertion |
| Postpartum | Valsalva manœuvres (incl. sneezing, coughing, laughing, defecation, vomiting) |
| **Concomitant pathologic conditions** | Acute emotion (e.g, fierce argument, acute anxiety) |
| Any surgical procedure | Bathing, showering, contact with water |
| Any cervical or cranial trauma | Other physiological trigger (e.g. mouvement, bending, micturition) |
| Cold or other ear-nose-throat infection |  |
| Any other infection |  |
| Other medical illness including pheochromocytoma, pre-/eclampsia,  intracranial hypotension |  |
| **Medications/illicit drugs**† |  |
| Cannabis |  |
| Other illicit drugs, including amphetamines, ecstasy, cocaine/crack,  lysergic acid diethylamide (LSD) |  |
| Selective serotonin reuptake inhibitors (SSRI) or serotonin–norepinephrine  reuptake inhibitors (SNRI) |  |
| Any other vasoactive medication, including adrenaline, local/epidural  anesthesia, triptans, ergot alkaloids, nicotine patch, methylphenidate,  nasal decongestants, steroids, immunosuppression/interferon, hormonal  treatment |  |

†Taken within four weeks of disease onset (except for medication/drugs with a longer half-life).
